# Structural insights into substrate selectivity of ribosomal RNA methyltransferase RlmCD

**DOI:** 10.1371/journal.pone.0185226

**Published:** 2017-09-26

**Authors:** Yiyang Jiang, Fudong Li, Jihui Wu, Yunyu Shi, Qingguo Gong

**Affiliations:** Hefei National Laboratory For Physical Sciences at Microscale and School of Life Sciences, University of Science and Technology of China, Hefei, Anhui, China; Universität Stuttgart, GERMANY

## Abstract

RlmCD has recently been identified as the S-adenosyl methionine (SAM)-dependent methyltransferase responsible for the formation of m^5^U at U747 and U1939 of 23S ribosomal RNA in *Streptococcus pneumoniae*. In this research, we determine the high-resolution crystal structures of apo-form RlmCD and its complex with SAH. Using an in-vitro methyltransferase assay, we reveal the crucial residues for its catalytic functions. Furthermore, structural comparison between RlmCD and its structural homologue RumA, which only catalyzes the m^5^U1939 in *Escherichia coli*, implicates that a unique long linker in the central domain of RlmCD is the key factor in determining its substrate selectivity. Its significance in the enzyme activity of RlmCD is further confirmed by in-vitro methyltransferase assay.

## Introduction

RNA methylation is a common and naturally-occurring event in both prokaryotic and eukaryotic organisms. It has been identified in many types of RNA molecules, including message RNA (mRNA), transfer RNA (tRNA), ribosomal RNA (rRNA), and non-coding RNA. Thus far, tRNA and rRNA have the most identified methylation modifications among all types of RNA molecules [1]. There are up to ~100 different types of modifications that occur in over 30 different tRNA positions, the majority being methylation [2]. Another kind of highly modified RNA molecule, rRNA, requires a number of significant methylations at its nucleobases or ribose for maturation [3]. For instance, 10 and 14 methylation have already been identified in the 16S and 23S rRNA of *E*.*coli*, respectively, with a variety of methylation types including m^1^G, m^3^U, m^5^U, m^5^C and m^2^A[1]. Almost all these methylated nucleotides are clustered at the functionally important sites of rRNA, such as the peptidyl transferase center (PTC), the nascent peptide exit tunnel (NPET), and the A, P, and E sites of tRNA binding sites, suggesting a strong connection between these modifications and the ribosomal function [4].

A prokaryotic ribosome is composed of a smaller 30S subunit and a larger 50S subunit, and 23S rRNA is a 2904-nt long component of the 50S subunit in *E*.*coli* [5]. During the process of protein translation, amino acids are first polymerized into the polypeptide chain in the ribosomal PTC. Newly synthesized polypeptides then extrude through the NPET, starting at the PTC and spanning the body of the 50S subunit, and finally leaving the ribosome [6, 7]. 23S rRNA plays an indispensable role in the above process. Two NPET regions associated with 23S rRNA have been proven to be essential in ribosome stalling, an important mechanism in the regulation of protein expression [7]. The first NPET region is composed of 23S rRNA nucleotides which form part of the so-called outer layer of PTC [8], while the second one is located at the constricted segment of NPET consisting of not only the 23S rRNA nucleotides, but also the amino acid residues from the ribosomal proteins L4 and L22 [9]. During translation, all nascent polypeptides must traverse and exit the NPET. The interactions of the nascent peptide chain with the exit tunnel can modulate the rate of protein synthesis, leading to pausing or stalling of translational elongation [10]. Some macrolide antibiotics that are able to bind within the exit tunnel can also induce the stalling of translation elongation, erythromycin being one of the representative drugs among them [9, 11–13].

Approximately one-third of modified residues in the 23S rRNA are clustered around the NPET, and one such heavily modified rRNA segment is the loop region of helix 35 in 23S rRNA [14]. Recent studies have revealed several important modifications within this loop in *Escherichia coli*, including m^1^G745, Ψ746, and m^5^U747 [14–16]. In gram-positive bacteria, however, m^1^G748 takes the place of m^1^G745 as a common methylation [17–19]. It has been indicated that these methylations interfere in the binding of macrolide and ketolide antibiotics to the ribosome [20]. The methylation at U747 seems to be a prerequisite for the m^1^G748 modification in gram-positive bacteria since U747 methylation has been suggested to promote the efficient G748 methylation by RlmA^II^ [17]. Auxilien et al. have indicated that RlmC is the specific methyltransferase (MTase) for the m^5^U modification at U747 in *E*.*coli* while RumA has been previously proven to be the m^5^U MTase for U1939 in gram-negative bacteria [14, 21]. However, it has been reported that, in *Bacillus subtilis*, the methylation of U747 and U1939 are both catalyzed by one same MTase YefA [22]. Recently, Shoji and coworkers also reported that, in *Streptococcus pneumoniae*, RlmCD functions as the MTase for both m^5^U747 and m^5^U1939 as well [17]. Currently, the specific catalytic mechanism and substrate selectivity of YefA or RlmCD remain elusive and underdetermined.

RNA MTases often use the S-Adenosyl methionine (SAM) as the methyl donor to catalyze the methyl group transfer to various positions of nucleotide base [23]. Thus far, all reported RNA MTases can be classified into at least four unrelated families, which are Rossmann-fold MTase (RFM) superfamily, SPOUT (SpoU-TrmD) superfamily, radical-SAM family and FAD/NAD(p)-binding protein family [24, 25]. The RFM is the largest MTase family, and it also contains the majority of DNA MTases [26]. SPOUT MTase superfamily is the second largest group, which exhibits an unusual α/β fold with a very deep topological knot [27, 28]. The SPOUT MTase members are dominantly dimers with the catalytic site formed at the interface of two monomers. In recent years, monomeric SPOUT MTase has been reported for tRNA m^1^G9 [2].

Here we report the crystal structures of RlmCD in the apo-form and in complex with SAH and an 18-nt RNA hairpin representing the helix 35 of 23S rRNA in *S*. *pneumoniae*. Our structural evidences indicate that RlmCD is a MTase that belongs to the RFM family. Using ^3^H-SAM as the methyl donor, we further perform in-vitro MTase activity assay of RlmCD for helix 35 of 23S rRNA and prove that RlmCD can methylate U747 specifically. Guided by the structural differences between RlmCD and its homologue RumA, we generate a series of mutants to investigate the contribution of a long linker existing in the central domain of RlmCD to its MTase activity, and implicate that this novel linker is the crucial factor for RlmCD to differentiate its different RNA substrates.

## Results

### RlmCD can specifically catalyze the methylation of U747 of 23S rRNA in vitro

**Fig 1:**
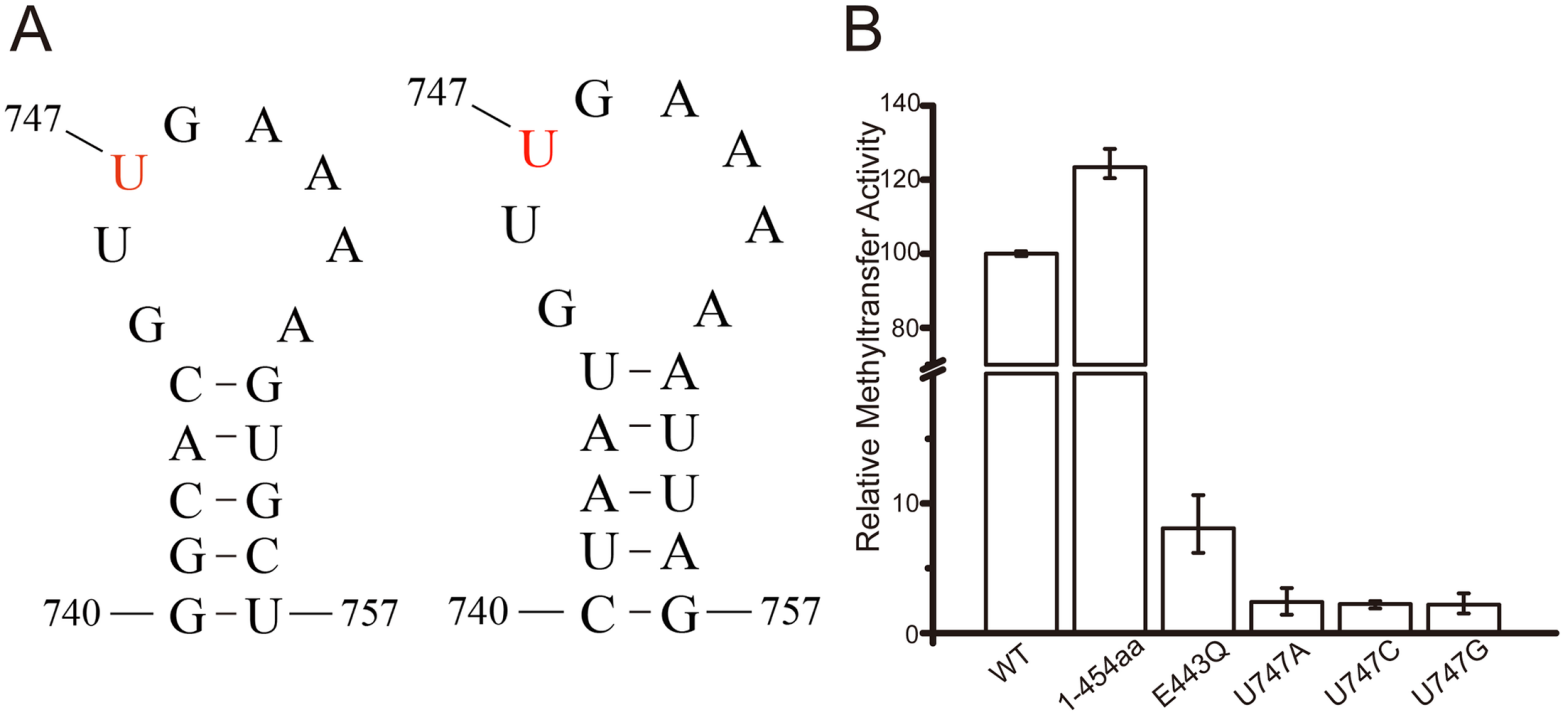
The 23S rRNA helix 35 is the substrate of RlmCD. (A) Secondary structures of the 18-mer RNA fragments of the *S*. *pneumoniae* (left) and *E*.*coli* (right) 23S rRNA helix 35. (B) In-vitro methyltransferase assay of RlmCD. The left three columns represent the methyl transfer activities of the wild-type RlmCD or its mutants toward rRNA-h35. The right three columns represent the methyl transfer activities of the wild-type RlmCD toward the different derivatives of rRNA-h35 (U747A, U747G, and U747C).

Shoji and coworkers have previously reported that RlmCD is a homologue to RlmC and it can mediate the formations of both m^5^U747 and m^5^U1939 in *S*. *pneumoniae* [17]. Using ^3^H-SAM as the methyl donor and an 18-mer RNA analogue of the 23S rRNA helix 35 (5’-^740^GGCACGUUGAAAAGUGCC^757^-3’, hereafter called rRNA-h35) as the substrate (Fig 1A), we systematically investigated the MTase activity of full-length RlmCD and its shorter construct RlmCDs [29] for U747 with an in-vitro methylation assay, in which the tritiated methyl group can be detected on the substrate RNA after a successful methyl transfer reaction. RlmCDs consisting of the residues 1–454 was constructed to represent the core region of RlmCD for its MTase activity as suggested by the sequence alignment with RumA (S1 Fig)[30]. As summarized in Fig 1B, our results showed that both full-length RlmCD and its shorter construct RlmCDs exhibit strong MTase activity when compared with the glutamine mutant of E443 whose equivalent residues in RumA and TrmA are proposed to be the general base for the enzymes (S1 Fig), indicating that RlmCD can catalyze the methylation of the rRNA helix 35 and RlmCDs alone is sufficient for its MTase activity. It is a little surprising that RlmCDs shows a MTase activity ~25% greater than full-length RlmCD, suggesting a moderate self-inhibitory effect from its C-terminal region. Furthermore, the methylation assay was applied to verify the substrate specificity of the methyl transfer by using three derivatives of 18-mer rRNA-h35 (U747A, U747C, and U747G). Our results clearly indicated, compared with the wild-type, the methyl transfer activities were completely abolished for all three derivatives even though there exist two more uridines (U746 and U754) in these RNA sequences (Fig 1A). All together, our in-vitro experiments confirmed that RlmCD is a 23S rRNA MTase specific for U747.

### Overall structure of RlmCD

To better understand the catalytic function of RlmCD for methyl transfer, X-ray crystallography was employed to determine its atomic-level structure. The RlmCDs construct used for crystallization was designed based on the sequence alignment with its homologue in *E*.*coli*, RumA, whose structure has been previously determined [21]. The structure of RlmCDs was solved by molecular replacement using the structure of RumA (PDB ID 1UWV) as the search model and finally refined to R_work_ and R_free_ values of 18% and 21%, respectively, at 1.8Å resolution (Table 1). In this structure, each asymmetric unit contains only one protein molecule.

**Table 1:** Data collection and refinement statistics.

Data collection statistics	RlmCDs	RlmCDs-SAH-U747
**PDB ID**	5XJ1	5XJ2
**Data collection**		
Wavelength (Å)	0.9776	0.9776
Space group	*P2*_*1*_	*P2*_*1*_
Cell dimensions		
*a*, *b*, *c* (Å)	46.45 101.77 63.04	63.37 94.93 164.16
*α*, *β*, *γ* (°)	90.00 110.11 90.00	90.00 95.91 90.00
Resolution range (Å)	38.59 1.75 (1.79–1.75)	39.52 2.85 (2.90–2.85)
*R*_merge_ (%)	11.0(25.0)	36.6(99.1)
*I/σI*	4.6(1.7)	7.2(2.1)
CC*/CC_*1/2*_	(0.941)/(0.795)	(0.872)/(0.614)
Completeness (%)	99.9(100)	99.9(99.7)
Redundancy	5.3(5.1)	4.4(4.3)
Wilson B-factor (Å 2)	22.9	31.7
**Refinement**		
Number of reflections (overall)	51160	43704
Number of reflections (test set)	1993	1999
*R*_work_/*R*_free_ (%)	17.5/20.2	19.7/23.6
Number of atoms		
Protein/Ligands/Water	3604/43/256	14221/319/14
*B*-factors (Å^2^)		
Protein/Ligands/Water	28.37/43.76/34.42	30.04/64.37/6.34
R.M.S. deviations		
Bond length (Å)	0.007	0.011
Bond angles (°)	0.889	1.741
Ramachandran plot (%)		
Favored/Allowed/Outlier (%)	98/2/0	95/4/1

Values in parentheses are for the highest resolution shell.

**Fig 2:**
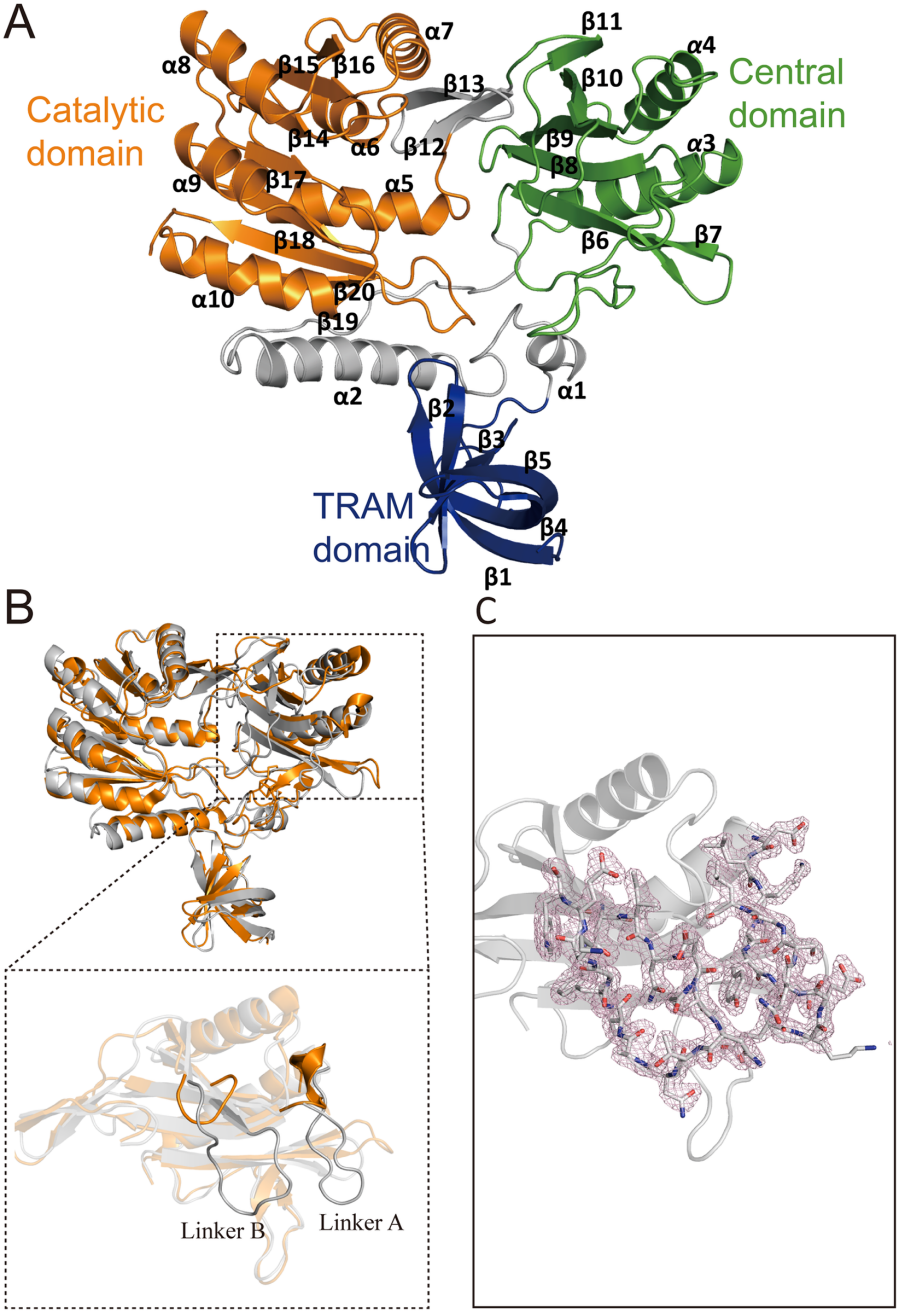
Overall structure of RlmCDs. (A) Three distinct parts of RlmCDs: the N-terminal TRAM domain, central domain, and C-terminal catalytic domain are colored in blue, green, and orange, respectively. The regions separating the three domains are all colored in grey. (B) The structure superimposition of RlmCDs and RumA (PDB ID 1UWV). RlmCDs and RumA are colored in gray and orange, respectively. (*Inset*) The superimposition of the central domain is individually shown to highlight the major difference between two structures. (C) The linker A and B of RlmCDs are shown in sticks as well as their electron density map with 2Fo-Fc calculated at 1σ.

**Fig 3:**
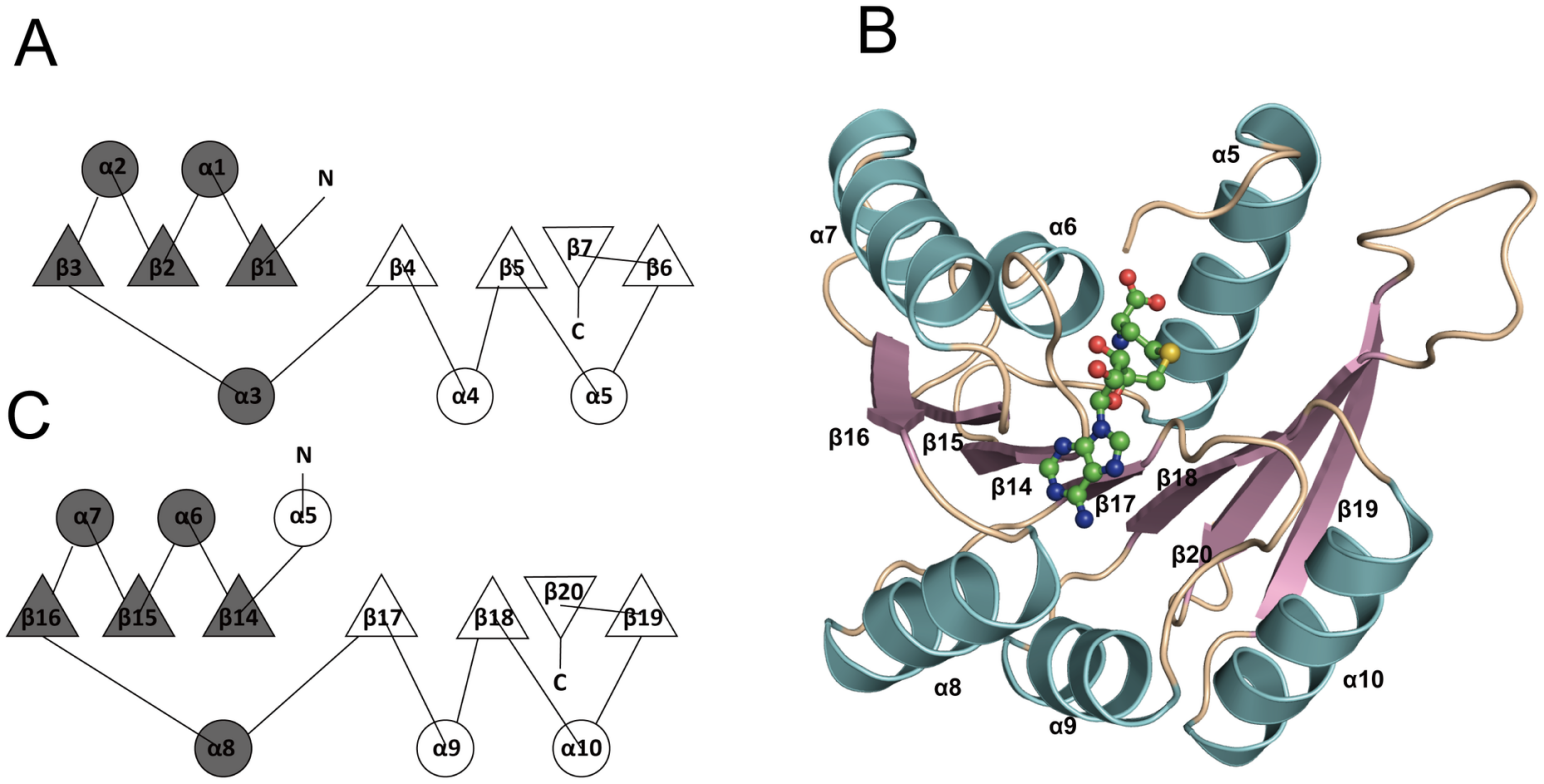
RlmCD belongs to the RFM family of MTases. (A) The canonical topology diagram of the catalytic domain in the RFM family of MTases. (B) Cartoon representation of the catalytic domain in RlmCD (residue 286–454). SAH is shown as ball-and-stick model. (C) The topology diagram of the catalytic domain in RlmCD. An extra α-helix (α5) is formed at the C-terminus of the catalytic domain.

The overall structure of RlmCDs highly resembles that of RumA with a RMSD of 1.9Å for the backbone Cα atoms. It is composed of three distinct domains representing the residues 1–63, 128–261, and 281–454, respectively (Fig 2A). The N-terminal TRAM domain is the smallest domain formed as a five-stranded antiparallel β-barrel (β1 to β5), which is also known as the typical OB fold. The central domain comprises a six-stranded β-sheet (β6 to β11) stacked against two α-helices (α3 and α4). The C-terminal catalytic domain exhibits a typical SAM-dependent MTase fold in which the β-strands (β14 to β20) of a seven-stranded β-sheet and α-helices (α5 to α10) are connected in an interleaved mode (Fig 2A). The structure of this catalytic domain is consistent with the consensus topological fold of RFM family MTases, except for having an extra α helix at its N-terminus (Fig 3B and 3C), indicating that RlmCD is a member of RFM family MTases.

To reveal whether there exist any structural differences between RlmCDs and RumA, we superimposed these two structures for both whole protein and individual domains (Fig 2B and S2 Fig). Most strikingly, compared with those in RumA, the linker regions between α3 and β8 (linker A) and between β10 and β11 (linker B) in the central domain are evidently longer in RlmCD (Fig 2B). These longer linkers are intrinsically interesting because the corresponding regions in RumA are directly involved in the crucial RNA recognitions in its complex structure with RNA substrate [31].

### Crucial residues participating in the SAM/SAH binding

To learn the structural basis for RlmCD-substrate binding specificity, we co-crystalized RlmCDs with SAM and 18-mer U747-methylated rRNA helix 35 in *S*. *pneumoniae* (5’-^740^GGCACGUm^5^UGAAAAGUGCC^757^-3’). In order to trap the complex in an inactive state favorable for crystallization, E443 in RlmCDs was mutated to glutamine (E443Q) as described in previous research [32]. The crystal structure of the ternary complex was finally determined at 2.85Å with R_work_ and R_free_ values of 20% and 24%. Different from the apo-form structure, each asymmetric unit of the ligand-bound form contains four molecules (protomers A-D) of the complex, in which the electron densities of SAH molecules, rather than SAM, in protomers A and C were well-defined while those in protomers B and D were partially observed. The cofactor in the complex structure was therefore identified as SAH, even though SAM was used for crystallization. On the other hand, the electron density of the RNA molecule can only be observed in one complex per asymmetric unit. A double-helical RNA representing the five base-pairs in the stem region of helix 35 can be fitted into this density whereas the loop region cannot be modeled due to the lack of the extra electron density (S3 Fig).

**Fig 4:**
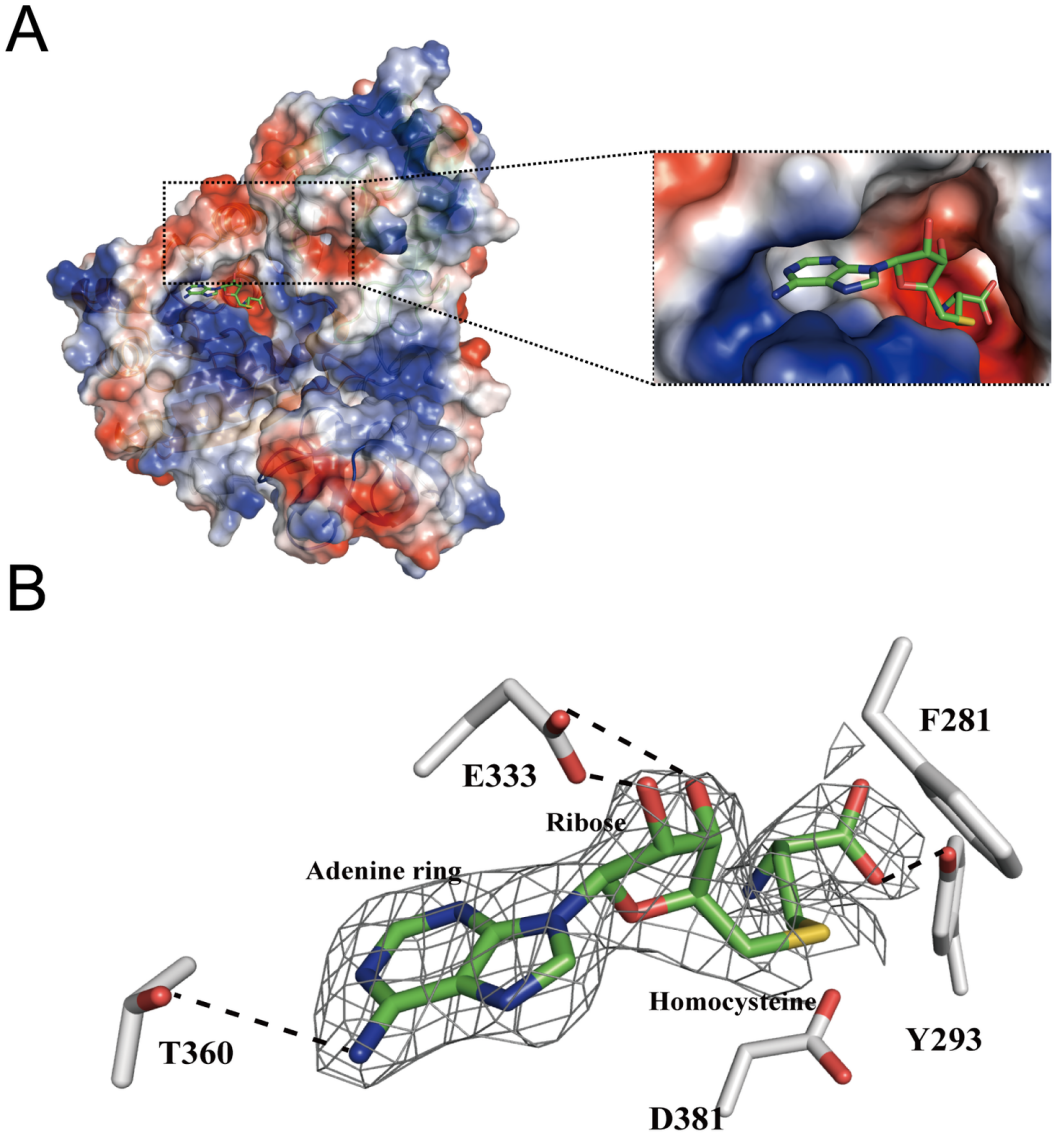
SAH binds RlmCD at a canonical binding pocket. (A) The overview of SAH anchored onto the catalytic domain of RlmCD. RlmCD is shown in its electrostatic surface potential, and SAH is shown as ball-and-stick model. (*Inset*) A close-up of the engagement of SAH into the binding pocket. (B) The interaction details of SAH with RlmCD. RlmCD residues are colored in gray and SAH is colored in green. The gray mesh represents 2Fo-Fc calculated at 1σ density map of SAH and the dashed lines represent the hydrogen bonds.

In its complex structure, the SAH molecule is enveloped in a deep pocket on the surface of the C-terminal MTase domain (Fig 4A) while the same pocket is also utilized to accommodate SAH in RumA [31]. The adenine ring and ribose of SAH lay down in the shallow area while the homocysteine part is entirely buried into the adjacent acidic pocket mainly constituted by the residues from α6 and α7, and the residues of the linker connecting α6 and β14 (Fig 4A). The detailed RlmCD recognition for SAH can be grouped into three moieties (Fig 4B). For the adenine ring of SAH, the N1 nitrogen forms two hydrogen bonds with the side-chain hydroxyl group of RlmCD T360. For the ribose moiety, the ribose hydroxyl of SAH makes a hydrogen bond with the γ-carboxyl group of RlmCD E333. Moreover, in the homocysteine moiety of SAH, the phenolic hydroxyl group of Y293 interacts with the homocysteine a-coo^-^ group via electrostatic attraction.

**Fig 5:**
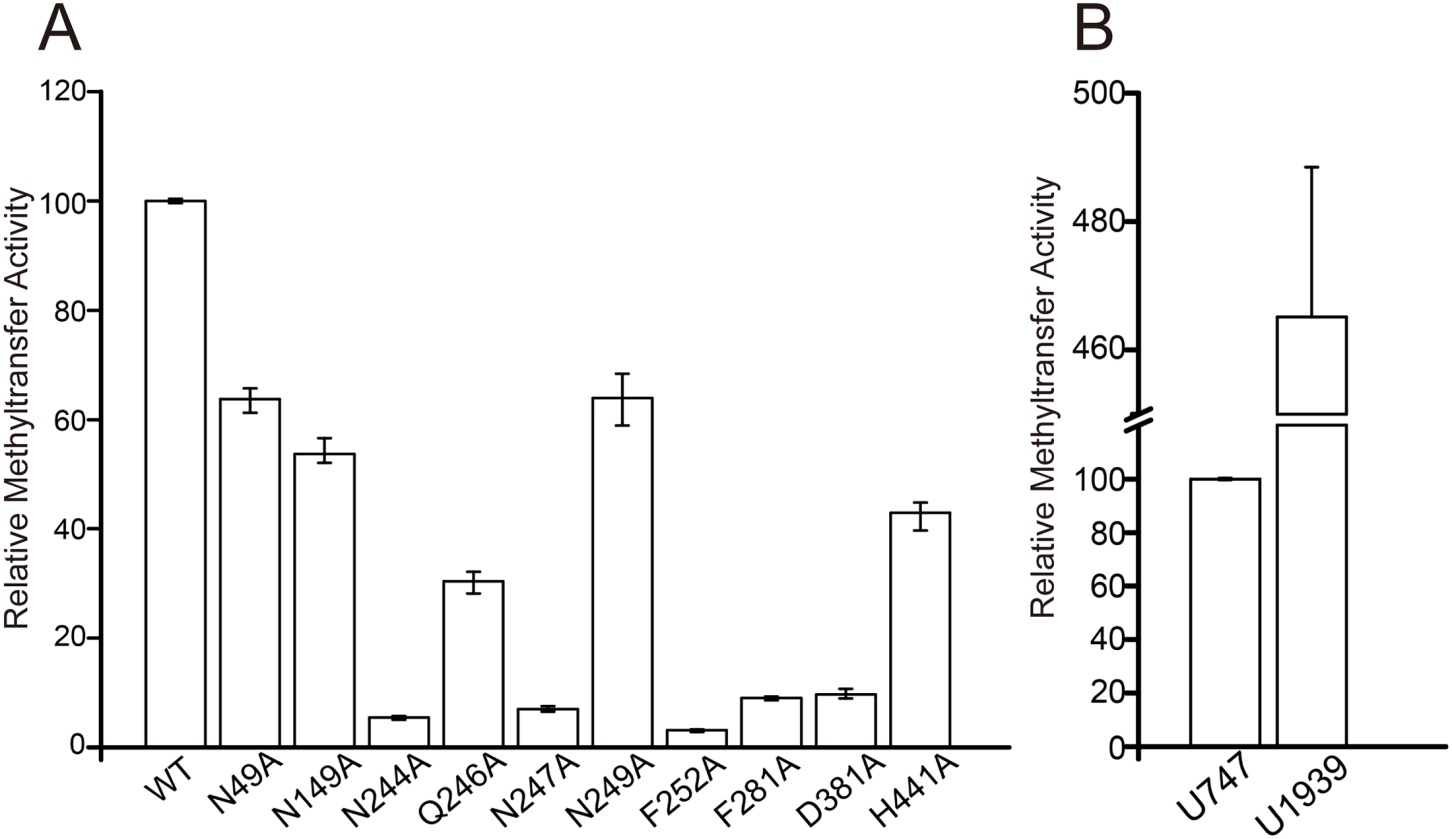
RlmCD is a 23S rRNA methyltransferase. (A) Comparison of the MTase activities of wild-type RlmCD and its mutants using rRNA-h35 as the substrate. (B) Comparison of the MTase activities of RlmCD toward U747 and U1939. The MTase activity of wild-type RlmCD was normalized to 100%.

A general structural characteristic of SAM-dependent MTase is that it always includes an aspartic acid or glutamic acid in its active site near the methyl group of the SAM [32]. In our complex structure of RlmCDs-SAH-RNA, residue D381 is in close proximity to the position of the methyl group in SAM (Fig 4B). To test whether this residue is crucial for the MTase activity of RlmCD, we generated a D381A mutant and investigated its MTase activity using in-vitro MTase assay. As expected, the activity of D381A reduced to nearly 10% of that of wide-type enzyme, implicating that this aspartic acid residue undertakes the same task as its equivalent residues in other SAM-dependent MTases (Fig 5A).

Moreover, in our complex structure, F281 is located at the entrance to the acidic pocket accommodating the homocysteine moiety of SAH molecule. Sequence alignment reveals, this phenylalanine is highly conserved in different rRNA MTases (S1 Fig). The MTase assay was therefore applied to investigate its contribution to the methyl transfer activity of RlmCD. Upon the mutation of F281 to alanine, RlmCD lost the majority of its MTase activity (Fig 5A), suggesting that F281 facilitates an efficient recognition for RlmCD to accommodate the methionine moiety of SAM using its canonical methionine-binding pocket.

Due to the short distance between these two residues and the position of the methyl group in SAM, ITC was further used to investigate the interactions of SAM with F281A, D381A, and wild-type RlmCD. Our results showed that both wild-type and mutants exhibit the comparable dissociation constants (K_d_) for SAM (S4 Fig), indicating that F281 and D381 don’t seem to affect the enzyme activity of RlmCD via the interaction with SAM.

### RlmCD uses a novel loop in its central domain to regulate the substrate selectivity

RlmCD is a recently identified MTase for ribosomal RNA in *S*. *pneumoniae*. Similar to its homologue YefA in *B*. *subtilis*, RlmCD can catalyze m^5^U methylation for both U747 and U1939 of 23S rRNA, whereas these two modifications are catalyzed by two different enzymes RlmC and RumA, respectively, in *E*. *coli*. In this study, we solved the crystal structures of RlmCD (RlmCDs) and its complex with SAH and RNA. Intriguingly, the structure of RlmCDs is very similar to that of RumA, raising the question of which crucial factor(s) determine the substrate selectivity in RlmCD when compared with RumA. Careful structural comparison between RlmCDs and RumA revealed the noticeable differences in the lengths of the two linker regions (liner A and B) in the central domains. These two linker regions both have 13 amino acids in RlmCD while their lengths in RumA are 3 and 6 residues, respectively. In the RumA-RNA structure, the central domain uses a consecutive basic patch to interact with a single-stranded region of 23S rRNA segment containing U1939 [31]. However, in RlmCD, this basic patch is largely perturbed by the long linkers (linker A and B) with residues mostly carrying no charge, suggesting a weakened RNA-binding capacity toward U1939-containing rRNA substrate for RlmCD (S5 Fig). On the other hand, our in-vitro MTase assay indicated that RlmCD shows an evident MTase activity toward U1939 even ~6-fold stronger than U747 (Fig 5B). To reconcile this apparent inconsistency, we propose that these linker regions may adopt the conformational changes to expose the RNA binding sites when interacting with the U1939-containing rRNA segment. This speculation is somewhat supported by the intrinsic flexibilities of these two linkers, which is suggested by the short side-chain and hydrophilic residue composition in these linkers (S5 Fig). In addition, considering RlmCD can catalyze the methylation transfer for both rRNA U747 and U1939 while RumA is a MTase only for U1939, we propose that the longer linkers are evolved in *S*. *pneumoniae* and *B*. *subtilis* to allow a relatively broader substrate selectivity for RlmCD.

Further sequence alignment revealed that, of these two linkers, linker B maintains a high degree of sequence conservation between RlmCD and YefA when compared with linker A, suggesting a possibility that linker B plays a more important role in RlmCD’s RNA-binding capacity and/or MTase activity (S1 Fig). To testify this hypothesis, several conserved polar residues and a phenylalanine within linker B were mutated to alanine (N244A, Q246A, N247A, N249A, and F252A) for MTase activity assay due to their potential in forming hydrogen-bonding or π-π stacking interactions. As summarized in Fig 5A, our results of MTase activity assay using rRNA-h35 as the substrate showed that the mutations of these residues caused the reductions in the MTase activity of RlmCD to different extents. Among them, N244A, N247A and F252A all exhibited dramatic experimental effects, retaining ~5%, ~6%, and ~7% of MTase activity of the wild-type, respectively, while other mutants (Q246A and N249A) caused only a moderate (~30%) and relatively minor (65%) effects (Fig 5A). Given that the crucial residues (F281 and D381) in the active site of MTase domain caused only ~90% reduction in the MTase activity of RlmCD, our results sufficiently proved that the certain residues of the linker B do play key roles in promoting an efficient MTase activity for RlmCD, probably through making important interactions with single-stranded RNA. Note that linker B also exists in RlmC (S5 Fig), but its residues are not conserved compared with RlmCD and YefA. The mechanism it uses for substrate selectivity remains to be illustrated. All mutants mentioned above were examined by CD to ensure the proper folding (S6 Fig).

## Discussion

In this research, through co-crystallization with SAM and an 18-mer RNA analogue to 23S rRNA helix 35, a complex structure of RlmCD E443Q was obtained. However, poor electron density of RNA molecule and its low abundance in one asymmetric unit raised the question of whether the interaction between RlmCD and RNA in this structure is specific and biologically significant. As the structural homologue to RlmCD, RumA has been previously investigated for its recognition of RNA substrate. In its complex structure with an 30-mer RNA segment of 23S rRNA (1932–1961), RumA uses the groove constituted by its central and catalytic domains to accommodate the 5’-end U1939-containing loop with A1937 and U1939 flipped out of the loop to interact with the MTase activity center of the catalytic domain, while the 3’-end hairpin segment of the RNA binds RumA in the cleft formed between the N-terminal TRAM domain and the catalytic domain with very few interactions observed between the stem of the hairpin and the protein. In our complex structure, although the quality of the electron-density map corresponding to the RNA is low, a double-helical RNA representing the five base-pairs in the stem region of helix 35 can be fitted without too much difficulty but the loop region including U747 can’t be modeled due to absence of excessive electron density. This double-helical RNA binds the cleft between the N-terminal TRAM and the catalytic domains of RlmCD in a similar mode as in RumA-RNA complex structure. Given that U747 is only three nucleotides away from the last base-pair of the stem (C744-G753), it is geometrically unlikely that U747 is able to reach the active center of the catalytic domain of RlmCD in our structure, indicating no possibility that it is a biologically important interaction. Besides, no obvious specific interactions were observed between the RNA and RlmCD, suggesting that the RNA presence in our crystal structure is likely to be the result of crystal packing. Even though, a similar binding mode for RNA duplex in the two complex structures of RlmCD and RumA implicates that the cleft between the TRAM and catalytic domains of RumA-like proteins has an optimal space to localize the double-helical RNA. In addition, the sequence alignment in the S1 Fig shows that the crucial residues participating into the recognition of U1939 are highly conserved among the m^5^U MTases of 23S rRNA, suggesting that RlmCD utilizes a similar recognition mode for U1939-containing RNA substrate as RumA.

Interestingly, as the MTase for m^5^U747 in *E*. *coli*, RlmC has a similar catalytic domain and central domain as RlmCD or RumA but lacks the N-terminal TRAM domain (S1 Fig). Given that the helix 35 of 23S rRNA is highly similar in *S*. *pneumoniae* and *E*. *coli* (Fig 1A), we speculate that the TRAM domain is not the necessary unit for RlmCD to maintain MTase activity for m^5^U747. To further study the structural basis of the catalytic mechanism RlmCD employed for m^5^U747 formation, we may need to either re-design the RNA substrate or weaken the nonspecific binding for RNA duplex by the TRAM and catalytic domains of RlmCD.

An iron-sulfur cluster coordinated by four conserved cysteine residues has been previously found in RumA and other RNA MTases [21]. Although the functional roles of iron-sulfur cluster in these RNA-modifying proteins remain unclear, its contribution to the conformational stability of these proteins has been suggested [31]. In the RumA-RNA complex, three cysteine (Cys81, Cys87, and Cys90) from an extend loop and Cys161 in the central domain constitute the binding pocket for iron-sulfur cluster, stabilizing the local conformation and forming a water-mediated hydrogen bond between its Gly89 and RNA substrate. Given that the overall structures of RumA and RlmCD are highly similar and that they both exhibit MTase activity for U1939 in 23S rRNA, it is quite interesting that RlmCD doesn’t possess an iron-sulfur cluster as RumA, but instead that the corresponding region in RlmCD (residues 72–81) folds as a short α-helix. The absence of the conserved cysteine residues and iron-sulfur cluster in RlmCD and some other 23 rRNA MTases [33] rules out the possibility that iron-sulfur cluster directly participates in the catalysis of the methyl transfer reaction. On the other hand, it is very likely that the well-folded α-helical region takes the place of iron-sulfur cluster in stabilizing the structure of the central domain. As a matter of fact, Gly89 of RumA and its equivalent residue in RlmCD, Gly79, maintain a similar conformation in two proteins (S7 Fig), suggesting that Gly79 may also participate in the interaction between RlmCD and U1939-containing RNA substrate. In summary, iron-sulfur cluster is an evolutionary ancient marker in sustaining the fundamental life processes in all living organisms. The presence/absence of this prosthetic group in two structural- and functional-related RNA-modifying proteins (RumA and RlmCD) may reflect an evolution process between *E*.*coli* and *S*. *pneumoniae* in which a metal-independent structural unit is evolved to fulfill the function due to environmental changes.

## Materials and methods

### Plasmid and RNA sample preparation

DNAs encoding full-length RlmCD were amplified from *Streptococcus pneumoniae* genome, then sub-cloned into pET28a modified plasmid (Novagen) which contains a 8*His-sumo tag and a ULP1 cleavage site at N terminal. The plasmids were subsequently transformed into BL21 (Gold) cell strains. All the mutants were generated using PCR and MutanBEST kit (TaKaRa).

All the *S*. *pneumoniae* 23S rRNA helix 35 fragments, 5’-^740^GGCACGUUGAAAAGUGCC^757^-3’ (U747, wild-type), 5’-^740^GGCACGUm^5^UGAAAAGUGCC^757^-3’ (m^5^U747), 5’-^740^GGCACGUAGAAAAGUGCC^757^-3’ (U747A), 5’-^740^GGCACGUGGAAAAGUGCC^757^-3’ (U747G) and 5’-^740^GGCACGUCGAAAAGUGCC^757^-3’ (U747C) were purchased from TaKaRa bio inc. RNA were diluted with DEPC water to a final concentration of 1 mM. Dilutions were heated for 5 min at 98°C, then put on ice for 5min. RNA dilutions were stored at -80°C until further use.

### In-vitro RNA transcription

The 30nt U1939-containing 23S rRNA 5’-^1932^GCGAAAUUCCUUGUCGGGUAAGUUCCGACC^1961^-3’ was obtained by using in-vitro RNA transcription. The transcription primer is 5’-GAAATTAATACGACTCACTATAGCGAAATTCCTTGTCGGGTAAGTTCCGACC-3’ with a 22nt T7 promoter pair region at the 5’ site. The 10ml transcription mixture contains 10mM DTT, 5mM dNTPs, 5mM MgCl_2_, 300nM T7 promoter, 300nM Primer, 1mg T7 RNA polymerase, and was diluted in 40mM Tris, pH8.1 buffer. The mixture was incubated at 37°C for 4 hours before heating it to 70°C for 20 minutes to quench the reaction. The mixture was then added with 1ml 0.5M EDTA, 1ml 5mM NaCl and 25ml pre-cold ethanol to precipitate the RNA.

The RNA precipitate was first collected by centrifugation at 15000g for 30 minutes. After removing the supernatant, the pellet was dried and dissolved in 1.5ml DEPC water. RNA was then purified by electrophoresis on urea-containing denaturing polyacrylamide gels at 120W. The RNA was visualized by UV-shadowing, and excised from the gel. The RNA was further eluted using the Elutrap Electroelution System (Whatman) at 150 V overnight. The purified RNA was then washed with 2M NaCl, and then desalted and exchanged into DEPC to a final concentration of 1.6M.

### Protein expression and purification

For protein expression, cells were initially grown in LB medium at 37°C to OD_600_ = 1.0. After induction by addition of 0.2mM isopropyl β-D-1-thiogalactopyranoside (IPTG), cells were grown further at 16°C for 24h. Cells were finally collected and suspended with Ni-NTA bind buffer (20mM Tris-HCl, 2M NaCl, pH8.0). The suspending cells were mixed with RNaseA and lysed by sonication. The lysate was collected by centrifugation and purified through Ni-NTA column (Qiagen). The eluent was then mixed with ULP1 cleavage enzyme and dialysed against the storage buffer (20mM Tris-HCl, 200mM NaCl, pH8.0) at 10°C for overnight. The protein sample was further purified by Superdex 200pg (16/60) (GE healthcare), and then transferred into the storage buffer and concentrated to 1mM. Mutant proteins were expressed and purified under the identical conditions as those used for wild-type RlmCD.

### In-vitro methyltransferase assay

The MTase reaction mixture (40μl) consists of 50mM Tris-HCl pH8.0, 1mM spermidine, 3mM MgCl_2_, 1mM DTT, 50mM NH_4_Ac, 1μCi ^3^H-SAM, 0.5μM enzyme and 1μM RNA. It was incubated at 37°C for 2h before adding 100μl water-saturated phenol into the mixture to quench the reaction. After centrifugation at 15000g for 20min, the aqueous phase was carefully removed from the phenol phase to a new tube, and added with 40μl chloroform/isoamyl alcohol (24:1 v/v) to extract the RNA. The RNA was then precipitated by addition of three times the volume of cold ethanol. After 2h precipitation at -20°C, the supernatant was removed by centrifugation, and the RNA pellet was diluted with 10μl DEPC water. The enzyme activity was calculated by counting the numbers of ^3^H on RNA products. For each assay, 10μl RNA dilution was added into 5ml flash liquid (ULTIMA-FLOTM, PerkinElmer), and counted for 60 seconds by QuantaSmart ^™^ (PerkinElmer). All the MTase assay experiments were performed in triplicate.

### Crystallization and structure determination

The 6mg/ml apo-form RlmCDs was used for initial crystallization trial which was set up in 48-well plates with sparse matrix crystallization suites (Index, CrystalScreen, PEGIon, and Natrix from Hampton Research; Proplex and FootPrintScreen from Molecular Dimesion) using sitting drop vapor at 20°C. Protein was mixed with buffer in a 1:1 ratio to equilibrate against 100 μl reservoir solution. Primitive crystals were grown in 0.1M HEPESNa, 45% W/V PEG600, pH7.5. The crystals for data collection were finally grown under the condition of 0.1M HEPES, 0.2M NaCl, 44%PEG600, pH7.3, using hanging drop method. The crystals for diffraction were transferred into cryo-protectant supplemented with 30% V/V glycerol and flash froze into liquid nitrogen.

For the crystallization of the RlmCDs-SAH-RNA complex, the purified RlmCDs E443Q mutant was concentrated to 8mg/ml, then mixed with SAM and an 18-mer U747-methylated rRNA segment in a ratio 1:2:1.2, and incubated on ice overnight. The initial crystallization screening conditions were as same as those for apo-form RlmCDs. Crystals were finally grown at 0.1M Ammonium sulfate, 0.01M Magnesium chloride hexahydrate, 0.05M MES monohydrate, 20% W/V PEG8000, pH5.6.

X-ray intensity data of the crystals were collected on Beamline 18U1 at Shanghai Synchrotron Radiation Facility (SSRF). The initial data were indexed, integrated, and scaled by HKL2000 package. Considering RlmCDs shares a ~30% sequence identity with full-length RumA, the structure phase of apo-form RlmCDs was solved by molecular replacement using RumA (PDB ID 1UWV) as the search model. After initial diffraction data procession, the structure of apo-form RlmCDs was firstly solved by the molecular replacement method employing the program Molrep and using the model derived from the RumA structure without solvent molecules or cofactors. The structure of RlmCDs was finally determined by the PHASER program from the CCP4 package and using the Molrep result as the initial model. Initial rounds of manual model building were performed with COOT under the electron density map contoured at 1.0σ, and all subsequent rounds of refinement were performed using Phenix refine and coot interchangeably. The rmsd between full-length RumA (PDB ID: 1UWV) and RlmCDs is calculated on Pymol with 270 C-alpha atoms.

The diffraction data of RlmCDs-SAH-RNA complex were identically processed with the apo-form structure as the initial model.

### Isothermal titration calorimetry measurement

The wild-type RlmCD and its mutants (F281A and D381A) were purified as above described. A MicroCal iTC200 system (GE Healthcare) was used to conduct the ITC measurements. The final concentration of SAM used for ITC was 1.5 mM, while those of the wild-type RlmCD, F281A, and D381A mutants were 0.1 mM, 0.05mM and 0.07 mM, respectively. Protein and SAM are dissolved in storage buffer (20mM Tris-HCl, 200mM NaCl, pH8.0).Protein concentrations were determined based on their UV280 absorbance. All ITC experiments had 20 injections of 2 μl cofactor into 200 μl protein with a spacing time 120s. In a control experiment, the buffer without the cofactor was injected into protein to compensate for the heat of protein dilution. All ITC measurements were carried out at 16°C. The resultant ITC curves were processed with software ORIGEN 7.0 (MicroCal) using a one-site fitting model.

### Circular dichroism

Far-UV CD spectra of The wild-type RlmCD and its mutants were carried out on an Applied Photophysics Chriascan spectrometer at 20°C. The spectra were recorded at wavelength between 195 and 260 nm using a 0.05 cm path length cell. The protein samples were diluted to 0.1 mg/ml with the CD buffer (30mM sodium phosphate buffer, pH8.0). A buffer-only reference was subtracted from each curve. All samples were tested in triplicate.

## Supporting information

S1 Fig Sequence alignment of full-length *S*. *pneumoniae* RlmCD, *B*.*subtilis YefA*, *E*.*coli* RumA and *E*.*coli* RlmCD. The conserved residues are white on a red background, and the similar residues are red in a blue rectangle. The linker A and B are highlighted with the underline. The residues of the catalytic domain participating into the U1939 recognition in RumA-RNA structure are labeled with blue asterisk.
(TIF)

S2 Fig Structure alignments of the C-terminal TRAM domain, central domain, and N-terminal catalytic domain between RlmCDs and RumA. RlmCDs and RumA are colored in gray and orange, respectively.
(TIF)

S3 Fig Overall structure of the RlmCDs-SAH-RNA complex. RNA is an 18-mer RNA analogue of the 23S rRNA helix 35 depicted in Fig 1. Left: RlmCDs is shown in cartoon within its electrostatic surface; the double helix region of RNA is shown in cartoon. Right: The RNA is shown in sticks within its 2Fo-Fc electron density map calculated at 1σ.
(TIF)

S4 Fig ITC analysis of SAM binding of wild-type RlmCD and its mutants. (A) The titration and fitting curves of wild-type RlmCD (WT), F281A, and D381A. (B) The dissociation constants (K_d_) of the ITC experiments.
(DOCX)

S5 Fig The structural mimic of RlmCDs in complex with U1939-RNA. (A) The structure of RumA-SAH-RNA ternary complex (PDB ID 2BHR). RumA is shown in its electrostatic surface potential and RNA is shown in orange. (B) The replacement of RumA with RlmCDs (PDB ID 5XJ1) in the structure of the RumA-SAH-RNA complex reveals obvious steric collision between the RNA and long linker regions in the central domain.
(DOCX)

S6 Fig CD spectra of wild-type RlmCD and all the mutants involved in this research. (DOCX)

S7 Fig Structure alignment of the iron-sulfur cluster binding pocket of RumA with the corresponding region in RlmCDs. RumA is colored in orange and RlmCD is colored in gray. The iron-sulfur cluster is shown in stick model.
(TIF)
