# Supplementary figures and images for: Structural insights into substrate selectivity of ribosomal RNA methyltransferase RlmCD
Source: PLoS One. 2017 Sep 26;12(9):e0185226. doi: 10.1371/journal.pone.0185226 (PMC5614603; doi:10.1371/journal.pone.0185226)

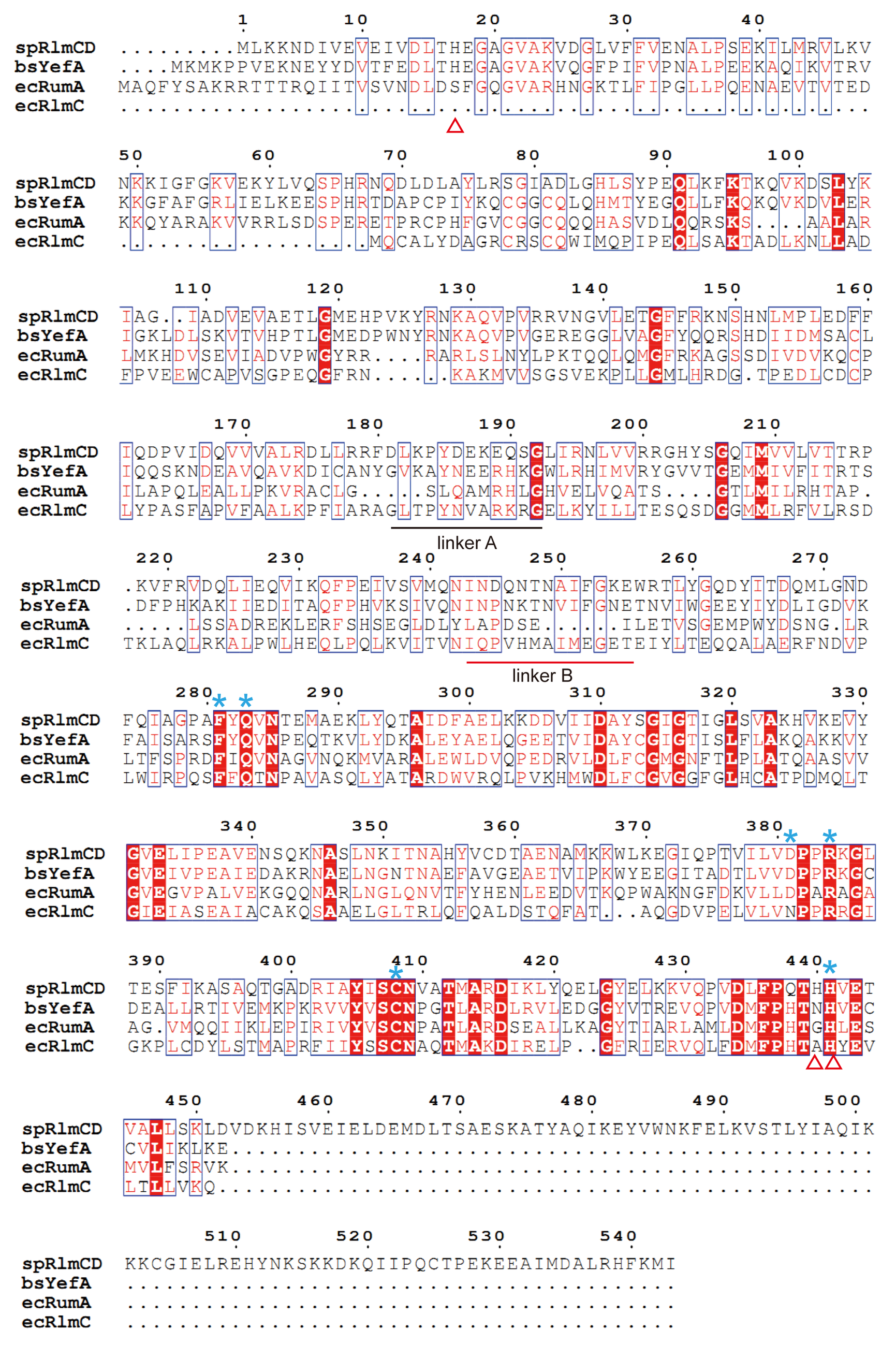

Supplement: S1 Fig — The conserved residues are white on a red background, and the similar residues are red in a blue rectangle. The linker A and B are highlighted with the underline. The residues of the catalytic domain participating into the U1939 recognition in RumA-RNA structure are labeled with blue asterisk. (TIF) [file pone.0185226.s001.tif]

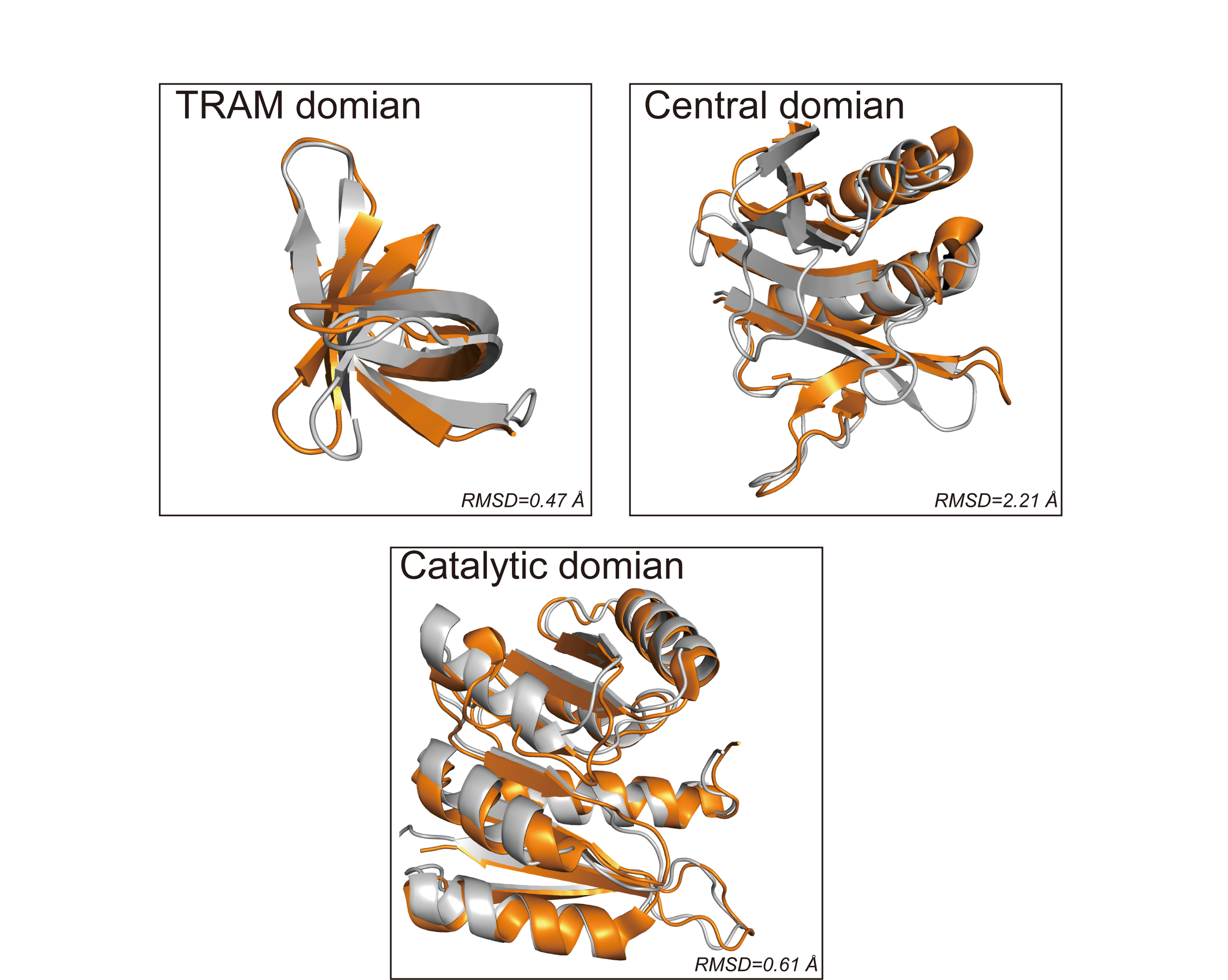

Supplement: S2 Fig — RlmCDs and RumA are colored in gray and orange, respectively. (TIF) [file pone.0185226.s002.tif]

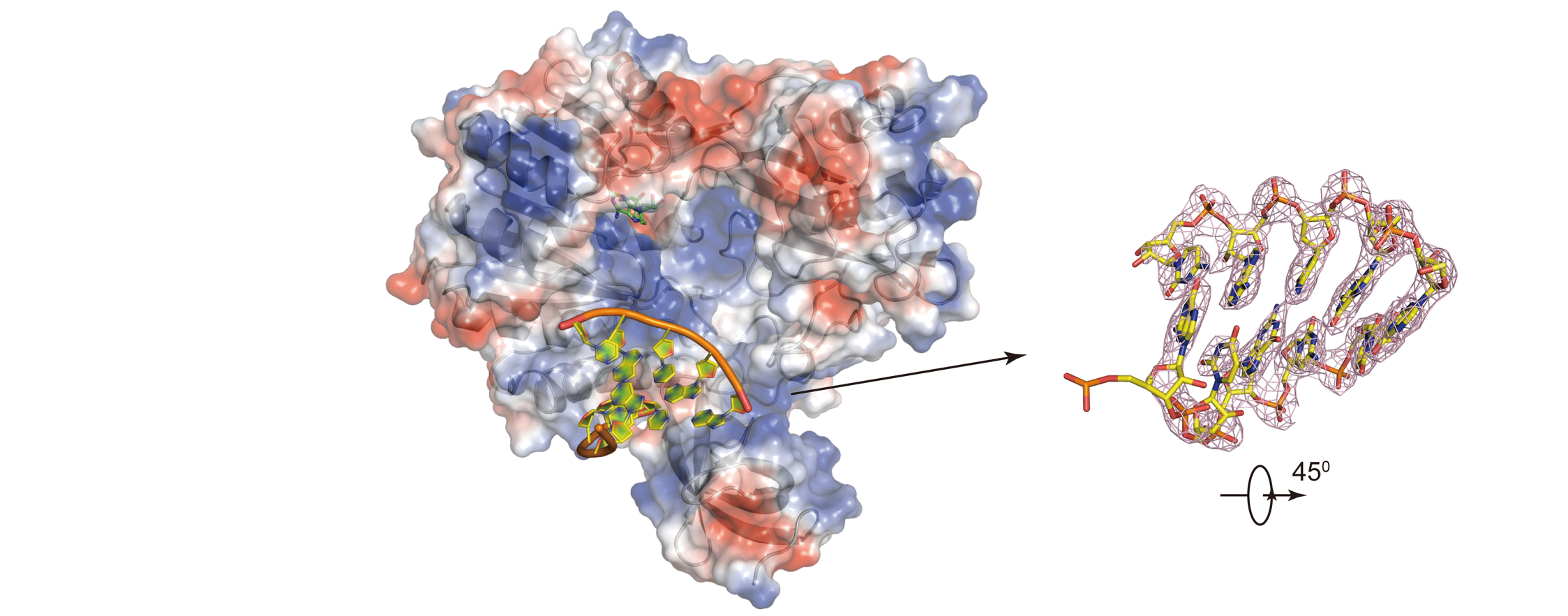

Supplement: S3 Fig — RNA is an 18-mer RNA analogue of the 23S rRNA helix 35 depicted in Fig 1. Left: RlmCDs is shown in cartoon within its electrostatic surface; the double helix region of RNA is shown in cartoon. Right: The RNA is shown in sticks within its 2Fo-Fc electron density map calculated at 1σ. (TIF) [file pone.0185226.s003.tif]

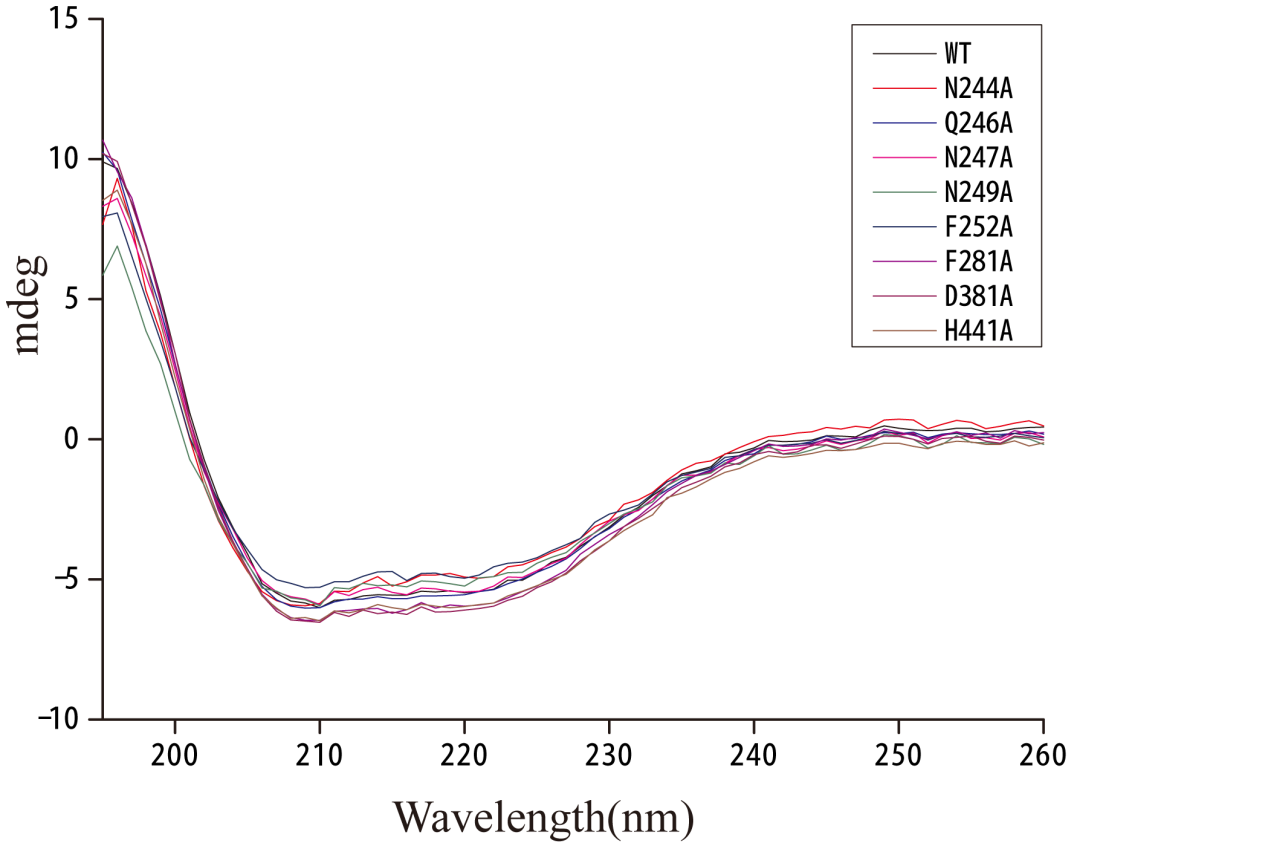


**S6 Fig.** CD spectra of wild-type RlmCD and all the mutants involved in this research.

Supplement: S6 Fig — (DOCX) [file pone.0185226.s006.docx]

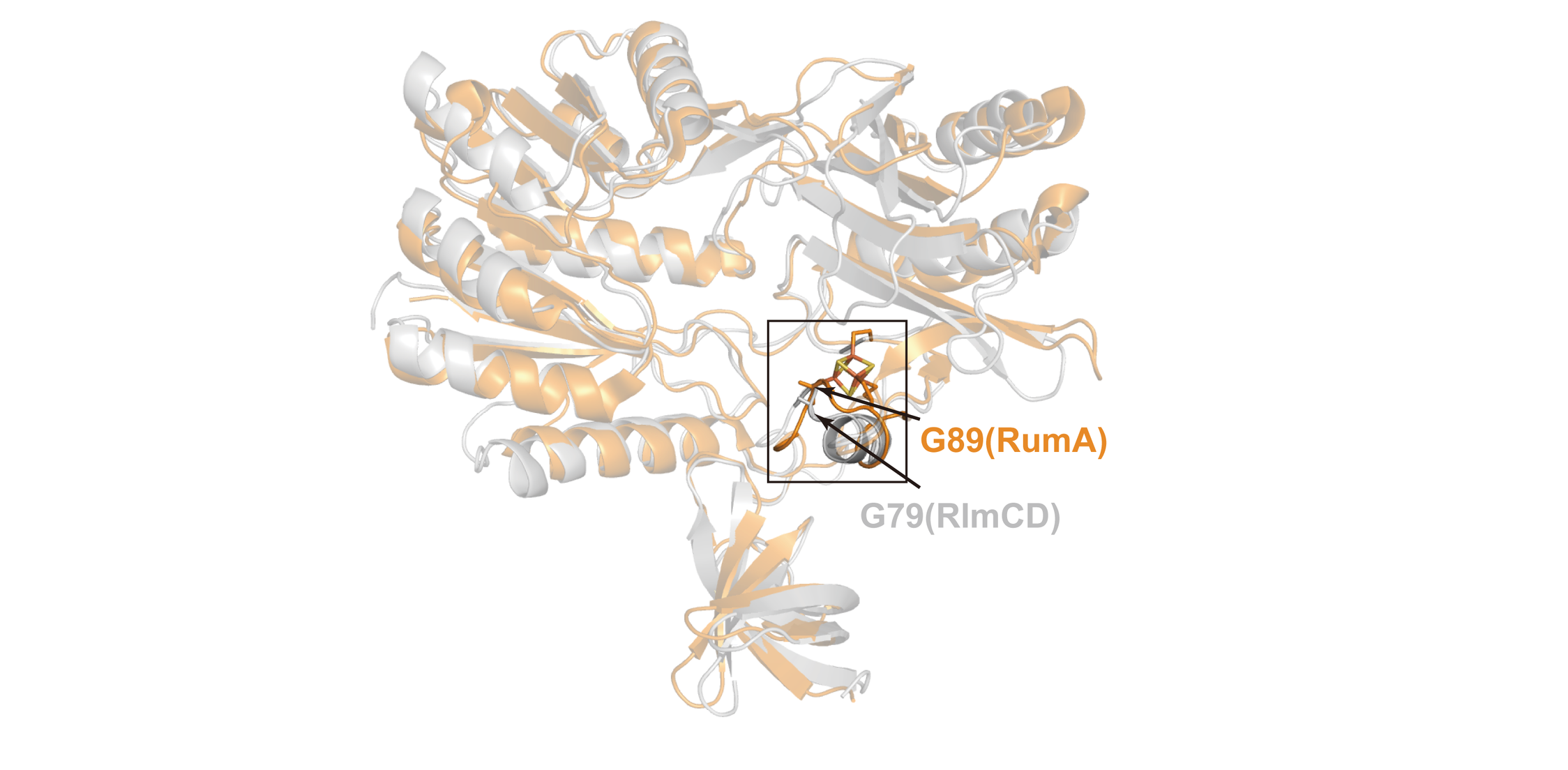

Supplement: S7 Fig — RumA is colored in orange and RlmCD is colored in gray. The iron-sulfur cluster is shown in stick model. (TIF) [file pone.0185226.s007.tif]
