# Supplementary material for: Structural insights into substrate selectivity of ribosomal RNA methyltransferase RlmCD
Source: PLoS One. 2017 Sep 26;12(9):e0185226. doi: 10.1371/journal.pone.0185226 (PMC5614603; doi:10.1371/journal.pone.0185226)
Supplement: S4 Fig — (A) The titration and fitting curves of wild-type RlmCD (WT), F281A, and D381A. (B) The dissociation constants (Kd) of the ITC experiments. (DOCX) [file pone.0185226.s004.docx]

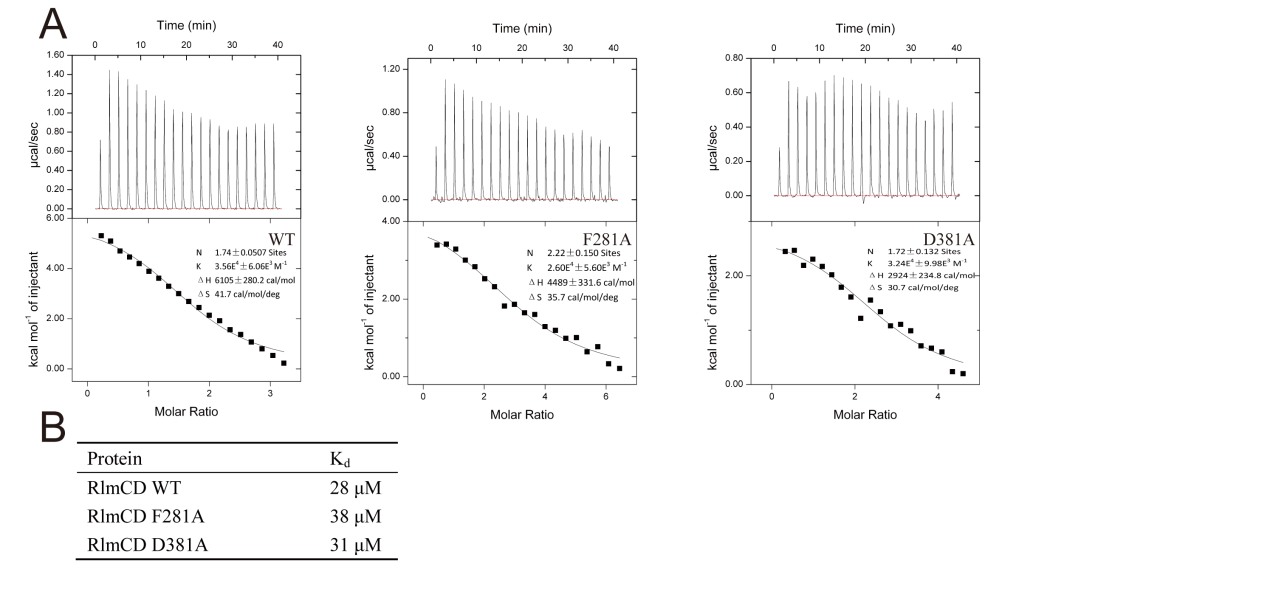


**S4 Fig. ITC analysis of SAM binding of wild-type RlmCD and its mutants.** (A) The titration and fitting curves of wild-type RlmCD (WT), F281A, and D381A. (B) The dissociation constants (K_d_) of the ITC experiments.
