# Supplementary material for: Structural insights into substrate selectivity of ribosomal RNA methyltransferase RlmCD
Source: PLoS One. 2017 Sep 26;12(9):e0185226. doi: 10.1371/journal.pone.0185226 (PMC5614603; doi:10.1371/journal.pone.0185226)
Supplement: S5 Fig — (A) The structure of RumA-SAH-RNA ternary complex (PDB ID 2BHR). RumA is shown in its electrostatic surface potential and RNA is shown in orange. (B) The replacement of RumA with RlmCDs (PDB ID 5XJ1) in the structure of the RumA-SAH-RNA complex reveals obvious steric collision between the RNA and long linker regions in the central domain. (DOCX) [file pone.0185226.s005.docx]

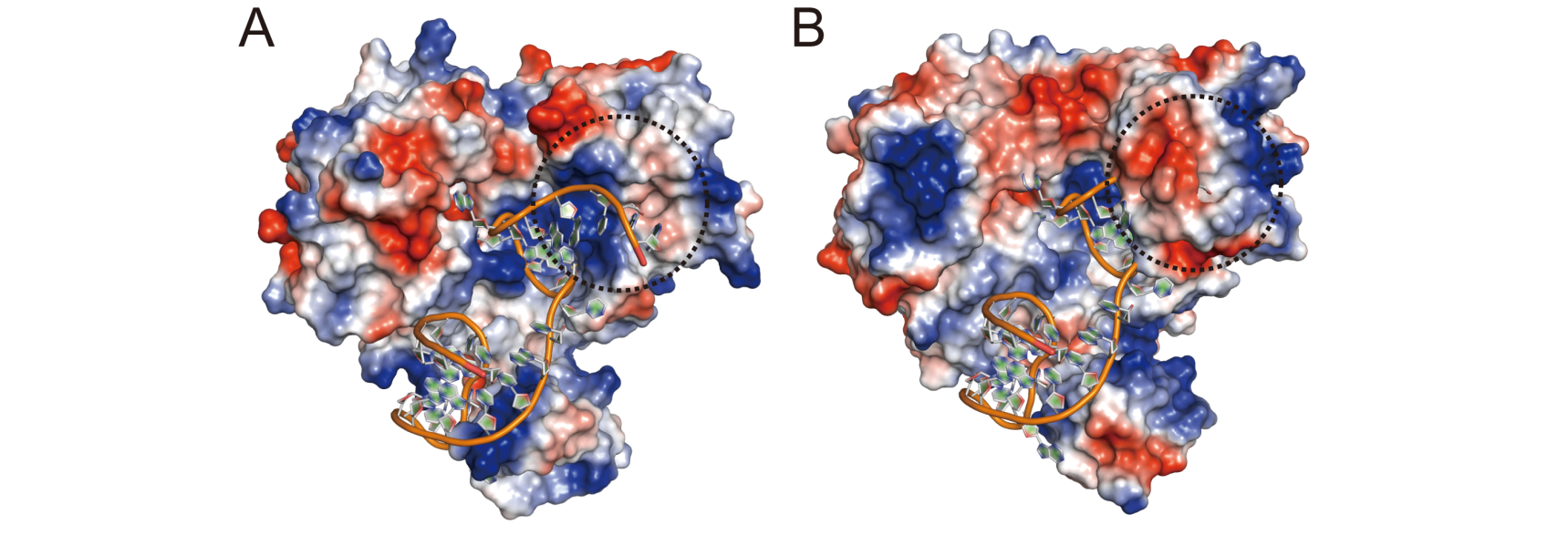


**S5 Fig. The structural mimic of RlmCDs in complex with U1939-RNA.** (A) The structure of RumA-SAH-RNA ternary complex (PDB ID 2BHR). RumA is shown in its electrostatic surface potential and RNA is shown in orange. (B) The replacement of RumA with RlmCDs (PDB ID 5XJ1) in the structure of the RumA-SAH-RNA complex reveals obvious steric collision between the RNA and long linker regions in the central domain.
